# Supplementary material for: A quantitative approach to measure and predict microbiome response to antibiotics
Source: mSphere. 2024 Sep 4;9(9):e00488-24. doi: 10.1128/msphere.00488-24 (PMC11423569; doi:10.1128/msphere.00488-24)
Supplement: Supplemental material — Figures S1-S3 and supplemental methods. [file msphere.00488-24-s0001.docx]

**Supplemental Figures**

**Figure S1. MiRIx applied to a previously published study of metronidazole.** Observed MiRIx values and taxonomic abundances for human vaginal microbiome study form Qin et al. Study shows 28 women with bacterial vaginosis where half of them responded to a 5-day metronidazole treatment with resolution of their symptoms and half of them didn’t respond with persistent symptoms.

**Figure S2. Relative abundance of antibiotic resistance genes in the study from Willmann et al.** Genes conferring resistance to fluoroquinolone antibiotics with a mean relative abundance of at least 10^-5^ are shown. Stars indicate a statistically significant difference relative to the abundance on day 0, after correction for multiple comparisons (*** P<0.001, ** P<0.01, * P<0.05).

**Figure S3. MiRIx applied to a previously published study of amoxicillin.** Observed MiRIx values and taxonomic abundances for human gut microbiome study form Dhariwal et al. Study shows before treatment of amoxicillin (n=8) or placebo (n=12), after treatment for three months and the follow up time point at 12 months.

**Supplemental Methods**

Here we describe the curation of the bacterial phenotype and antibiotic susceptibility databases and explanation of the MiRIx software in detail.

**Database curation:**

Antibiotics target various microbial genes to limit their growth. The bacteria that contain these targets can generally be summarized by their Gram staining or aerobic status (1). In order to categorize bacteria as resistant or susceptible to antibiotics of interest, we set out to curate a database of bacterial phenotypes. When a bacterial species is identified, the description and characteristics of the organisms is published in the International Journal of Systematic and Evolutionary Microbiology (IJSEM). Our group has done extensive literature search to curate Gram stain, aerobic status, morphology, motility, spore formation, bile sensitivity and saccharolytic fermentation ability of 798 species and 208 genera by combing through papers in IJSEM. Class and phylum level taxa are only annotated for aerobic status and Gram staining. These efforts are tabulated in the “taxon phenotypes” database.

Certain genera or species within the target group of an antibiotic can still be resistant or susceptible through inherent resistance genes. Therefore, we extracted information on bacteria that are susceptible or resistant to penicillin (2-6), tetracycline (3, 7-11), vancomycin (3, 12-16) and aminoglycoside (17) through literature search. These efforts are tabulated in the “taxon susceptibility” database.

The database is available from: https://github.com/PennChopMicrobiomeProgram/whatbacteria

**Software implementation:**

**Input:** Taxonomic lineage and the relative abundance of bacteria in a sample.

**Step 1) Searching annotation database:** Our software first annotates all the levels of taxonomy of the bacterial lineage using both the phenotype and susceptibility databases. Then it assigns the annotation for the most specific taxonomy level for that lineage.

**Step 2) Assign susceptible or resistant status:** There are dedicated functions to calculate susceptibility for four different classes of antibiotics based on their microbial target. For vancomycin, Gram-positive taxa as annotated as susceptible and Gram-negative taxa as resistant. Then our software looks through the antibiotics susceptibility database to identify the exceptions to this rule. For aminoglycosides, Gram-negative aerobes and Gram-negative facultative anaerobes are annotated as susceptible and the rest as resistant. Then our software looks through the antibiotics susceptibility database to identify exceptions to this rule. (3) For penicillin and tetracycline, all lineages are first annotated as susceptible as they are broad-spectrum antibiotics. Then our software looks through the antibiotics susceptibility database for known taxa that are resistant to the specific antibiotic.

**Step 3) Calculating the Microbiome Response Index:** Our software then finds the total relative abundance of susceptible and resistant taxa. The index is calculated by log 10 of the ratio between the relative abundances of susceptible to resistant bacteria.

**References**

1. Singh SB, Young K, Silver LL. 2017. What is an "ideal" antibiotic? Discovery challenges and path forward. Biochem Pharmacol 133:63-73.

2. Reygaert WC. 2018. An overview of the antimicrobial resistance mechanisms of bacteria. AIMS Microbiol 4:482-501.

3. Piddock LJ. 2006. Multidrug-resistance efflux pumps - not just for resistance. Nat Rev Microbiol 4:629-36.

4. Hollenbeck BL, Rice LB. 2012. Intrinsic and acquired resistance mechanisms in enterococcus. Virulence 3:421-33.

5. Cox G, Wright GD. 2013. Intrinsic antibiotic resistance: mechanisms, origins, challenges and solutions. Int J Med Microbiol 303:287-92.

6. Cilloniz C, Dominedo C, Torres A. 2019. Multidrug Resistant Gram-Negative Bacteria in Community-Acquired Pneumonia. Crit Care 23:79.

7. Waters JL, Salyers AA. 2013. Regulation of CTnDOT conjugative transfer is a complex and highly coordinated series of events. mBio 4:e00569-13.

8. Rudra P, Hurst-Hess K, Lappierre P, Ghosh P. 2018. High Levels of Intrinsic Tetracycline Resistance in Mycobacterium abscessus Are Conferred by a Tetracycline-Modifying Monooxygenase. Antimicrob Agents Chemother 62.

9. Margus T, Remm M, Tenson T. 2007. Phylogenetic distribution of translational GTPases in bacteria. BMC Genomics 8:15.

10. Grossman TH. 2016. Tetracycline Antibiotics and Resistance. Cold Spring Harb Perspect Med 6:a025387.

11. Davin-Regli A, Lavigne JP, Pages JM. 2019. Enterobacter spp.: Update on Taxonomy, Clinical Aspects, and Emerging Antimicrobial Resistance. Clin Microbiol Rev 32.

12. STANTON TB, SAVAGE* DC. 1983. Roseburia cecicola gen. nov., sp. nov., a Motile, Obligately Anaerobic Bacterium from a Mouse Cecum. International Journal of Systematic and Evolutionary Microbiology 33:618-627.

13. Nelson RR. 1999. Intrinsically vancomycin-resistant gram-positive organisms: clinical relevance and implications for infection control. J Hosp Infect 42:275-82.

14. Monticelli J, Knezevich A, Luzzati R, Di Bella S. 2018. Clinical management of non-faecium non-faecalis vancomycin-resistant enterococci infection. Focus on Enterococcus gallinarum and Enterococcus casseliflavus/flavescens. J Infect Chemother 24:237-246.

15. Martin R, Miquel S, Benevides L, Bridonneau C, Robert V, Hudault S, Chain F, Berteau O, Azevedo V, Chatel JM, Sokol H, Bermudez-Humaran LG, Thomas M, Langella P. 2017. Functional Characterization of Novel Faecalibacterium prausnitzii Strains Isolated from Healthy Volunteers: A Step Forward in the Use of F. prausnitzii as a Next-Generation Probiotic. Front Microbiol 8:1226.

16. Campedelli I, Mathur H, Salvetti E, Clarke S, Rea MC, Torriani S, Ross RP, Hill C, O'Toole PW. 2019. Genus-Wide Assessment of Antibiotic Resistance in Lactobacillus spp. Appl Environ Microbiol 85.

17. Germovsek E, Barker CI, Sharland M. 2017. What do I need to know about aminoglycoside antibiotics? Arch Dis Child Educ Pract Ed 102:89-93.
